# SEG-FAUSP: Anatomical Structure Segmentation of the Standard Sections of Fetal Abdominal Ultrasounds

**DOI:** 10.3390/bioengineering13040403

**Published:** 2026-03-31

**Authors:** Jianhui Chen, Peizhong Liu, Xiaying Yang, Xiaoling Wang, Xiuming Wu, Zhonghua Liu, Shunlan Liu

**Affiliations:** 1 College of Engineering, Huaqiao University, Quanzhou 362021, China; cjh545@163.com (J.C.); pzliu@hqu.edu.cn (P.L.); 2 Department of Ultrasound, The Second Affiliated Hospital of Fujian Medical University, Quanzhou 362000, China; 19942911457@163.com; 3 Department of Ultrasound, Quanzhou First Hospital Affiliated to Fujian Medical University, Quanzhou 362000, China; hxp1113@163.com (X.W.); wxming1981@163.com (X.W.)

**Keywords:** fetal abdominal ultrasound, organ segmentation, deep learning

## Abstract

This study addresses the challenge of the difficult identification of organ structures in the standard sections of fetal abdominal ultrasounds. A deep learning-based multi-task model named SEG-FAUSP was developed to segment the core anatomical structures of seven key fetal abdominal ultrasound sections. We collected fetal abdominal ultrasound images from pregnant women in the mid-pregnancy period (18–24 weeks) using various mainstream ultrasound devices, and professional physicians annotated key anatomical structures (e.g., umbilical veins, gastric bubbles, spine) in the images. Based on an improved deep learning framework, the model accurately segments and locates the target organ structures through a parallel dual-branch semantic segmentation network, which avoids the over-reliance on large-scale pre-trained data in traditional methods. Experimental results show that the model achieves excellent performance in anatomical structure segmentation, with the intersection over union of the bladder and gastric bubble both reaching above 0.84; its segmentation accuracy for complex structures such as the inferior vena cava is also significantly superior to the baseline model. As an end-to-end model, it simplifies the clinical interpretation process of fetal abdominal ultrasound, reduces the risk of missed diagnoses caused by unclear organ identification, provides an efficient auxiliary tool for prenatal screening in grassroots medical institutions, and is of great significance for improving the quality of newborns.

## 1. Introduction

According to recent global reports [1], neonatal congenital defects have become the fourth leading cause of neonatal death. Approximately 8 million infants are born with congenital defects each year, with the problem being particularly prominent in developing countries. Fetal abdominal malformation is one of the important causes of neonatal death [2]. The second trimester of pregnancy (20–26 weeks) is a critical window period for fetal structural malformation screening. At this stage, timely detection of abnormalities by ultrasound can reserve sufficient time for clinical intervention, otherwise, it may delay diagnosis and treatment and increase the risk of neonatal death [3].

With the continuous development of science and technology, medical imaging technology has continuously improved and has been widely used in clinical practice. Unlike traditional biopsy, this technique is non-invasive and can image internal tissues and organs, especially suitable for observing parts that are difficult to detect by the naked eye. Medical imaging techniques include X-ray examination, CT scanning, MRI examination and ultrasound examination.

Ultrasound technology takes advantage of the reflection and propagation of high-frequency sound waves in human tissues and generates dynamic images in real time by receiving echo signals. Compared with other medical imaging technologies, ultrasound imaging has advantages such as the absence of ionizing radiation, simple operation, and low cost. With the core advantages of safety and immediacy, this technology is widely used in obstetric monitoring, heart disease diagnosis and abdominal examination, and provides accurate guidance for interventional treatment.

A fetal abdominal ultrasound is the core link of prenatal screening, and its full clinical value depends on the accurate acquisition and standardized interpretation of standard sections. As the basic and core standard section, the transverse section of fetal abdominal circumference should clearly show the key anatomical structures such as the intrahepatic segment of umbilical vein, inferior vena cava, gastric bubble, abdominal aorta, and spine. It not only provides an important basis for the measurement of abdominal circumference and the assessment of fetal growth and development, but also can assist in the diagnosis or suggestion of permanent right umbilical vein, esophageal atresia, situs inversus and other abnormalities. The combined evaluation mode of multiple sections can further improve the efficiency of deformity detection, especially in the diagnosis of complex malformations, and the addition of relevant sections can significantly reduce the risk of missed diagnosis. As the preferred imaging method for screening fetal abdominal congenital malformations such as digestive, urinary and abdominal walls, a fetal abdominal ultrasound has the advantages of safety and non-invasiveness, which provides core support for clinical decision-making, and is a key technical means to ensure the health of mother and child and improve the quality of the birth population.

However, the objective existence of ultrasound artifacts seriously limits the accuracy of diagnosis. Such artifacts are caused by the physical characteristics of the sound beam propagation, the performance of the probe and equipment, the differences in the anatomical structure of the human body, and the operator’s operation specification, which will directly affect the quality of the ultrasound image and interfere with the accurate judgment of the location, size, shape, nature and range of the lesion. This not only increases the difficulty of diagnosis and prolongs the examination time, but also may lead to misdiagnosis and misjudgment, which in turn affects the formulation of treatment plans and increases medical risks [4].

More importantly, the acquisition and interpretation of standard sections rely heavily on physician experience. Different levels of professional physicians may lead to insufficient standardization of the sections, which directly affects the accuracy of diagnosis. To address this challenge, we propose the SEG-FAUSP model, which utilizes a multi-task deep learning framework to provide a basis for determining standard sections by precisely segmenting key anatomical structures. The rest of this paper is structured as follows: Section 2 systematically introduces the related field research; Section 3 focuses on the materials and methods used in this study. Section 4 focuses on the analysis of the experimental process and results. In Section 5 and Section 6, the research is discussed and the final conclusions are given. Overall, our main contributions include: (1) the construction of the FAUSP dataset based on the ISUOG standard, which contains 4010 images of the seven most frequently used basic planes in fetal abdominal ultrasound scans, filling the gap of high-quality labeled data for multi-plane segmentation of fetal abdominal ultrasounds; (2) a novel deep learning multi-task framework SEG-FAUSP is proposed for automatic recognition of FAUSP images and precise segmentation of key anatomical structures, which innovatively integrates ASPP, MSCA, BiFormer and CCA on the basis of DeepLabv3+ with a task-driven hierarchical feature fusion design for fetal abdominal ultrasound characteristics. This framework realizes the organic coupling of multi-scale feature extraction, long-range dependency modeling and contextual attention interaction, and fundamentally solves the problems of low segmentation accuracy of small and complex structures and poor feature representation of scattered anatomical layout in fetal abdominal ultrasound images.

## 2. Related Work

Recent advancements in deep learning have significantly impacted the segmentation and analysis of medical ultrasound images, particularly in the context of fetal ultrasound imaging. Xiao et al. [5] summarized the latest methods for ultrasound image and video segmentation, covering innovations such as diffusion models, weakly supervised learning, and the “Segment Anything” model, while addressing the challenges faced in clinical applications and outlining future research directions. In the realm of semantic segmentation, Li et al. [6] introduced the DCD model for fetal four-chamber (A4C) view ultrasounds, combining Dense-ASPP and attention modules to improve multi-scale feature representation, showing exceptional performance in fetal heart structure segmentation. Zhou et al. [7] proposed a multi-task deep learning framework integrated with radiomics for fetal ultrasound image classification and segmentation, demonstrating the effectiveness of multi-task learning in enhancing segmentation accuracy and aiding clinical diagnosis. To tackle the problem of limited annotated data, Wang et al. [8] developed the Dual Agreement Consistency Learning framework, which leverages pre-trained foundation models and collaborative training to improve segmentation performance for fetal heart ultrasound images. Moreover, Bai et al. [9] have pushed forward the standardization of fetal ultrasound segmentation, offering a dataset that covers multiple standard planes for comprehensive algorithm evaluation, thereby enhancing generalization. These studies collectively contribute to overcoming challenges such as image quality variability and complex anatomical structures, providing a robust foundation for the future clinical application of automated fetal ultrasound analysis.

In order to reduce the workload of sonographers and improve the accuracy, efficiency and interpretability of prenatal ultrasound standard plane detection, Zhao et al. [10] proposed a fetal head ultrasound standard plane detection (USPD) model based on multi-task learning and a hybrid knowledge map. In order to control the quality of fetal ultrasound (FS) images and improve the accuracy of abnormal fetus diagnoses, Zhang et al. [11] proposed an automatic image quality assessment scheme based on multi-task learning to efficiently assist sonographers in FS image quality control. In order to effectively help doctors distinguish normal and abnormal fetal brain ultrasounds in the standard axial plane, Xie et al. [12] used a deep learning algorithm to classify normal and abnormal fetal brain ultrasound images in the standard axial plane, and the algorithm had high accuracy and specificity in classification and lesion localization. In order to solve the problem that the traditional thyroid ultrasound standard plane (TUSP) classification method is time-consuming and laborious, which is easily affected by the subjective factors of doctors, and to meet the needs for automatic classification methods in clinical diagnosis, Guo et al. [13] compared CNN deep learning convolutional neural network models with different structures. An 18-layer ResNet model was used to automatically classify TUSP images. In order to solve the problem that manual acquisition of fetal ultrasound standard sections is time-consuming and laborious, and depends on the professional knowledge of the operator, and to deal with the challenges of large intra-class differences, small inter-class differences and low image quality of standard sections in ultrasound videos, Chen et al. [14] proposed a general convolutional recurrent neural network composite framework. Multi-class standard sections were automatically identified by the joint learning of intra-plane and inter-plane features. A multi-task learning framework was used to mine the common knowledge of different standard section detection tasks to alleviate the limitation of insufficient training data. In order to solve the problems of overfitting and accuracy degradation caused by the difficult detection of fetal brain tissue features and the scarcity of labeled data in the recognition of fetal brain ultrasound standard sections (FBSPs), Qu et al. [15] proposed a differential convolutional neural network (Differential-CNN). By applying the differential operator on the original CNN feature map to generate the differential feature map, the pixel directional pattern analysis was used to improve the automatic recognition performance of six types of FBSPs without increasing the number of convolutional layers and parameters.

In order to solve the difficult problem of prenatal diagnosis of fetal congenital heart disease (CHD), Wu et al. [16] proposed an automatic recognition method based on Fast R-CNN deep learning framework, which assisted sonographers with different experience levels to improve the efficiency of image acquisition and diagnostic reliability by efficiently identifying the standard section of a fetal heart. In order to achieve intelligent analysis of fetal ultrasound video streams, Pu et al. [17] proposed a fetal ultrasound standard section recognition (FUSPR) model deployed on a high-performance computing platform based on deep learning. The model learns spatial anatomical features and inter-frame timing information through CNN-RNN double-branch architecture. The feature fusion strategy is introduced to enhance the modeling ability of the spatial sequence and motion representation in the video stream. In order to achieve standardized abdominal circumference (AC) section detection without real-time eye movement data input while keeping high clinical accuracy, Cai et al. [18] proposed a novel multi-task convolutional neural network called Multi-task acoustic eye Movement Network (M-SEN). In order to solve the problem that the recognition process of fetal ultrasound standard section is complex, subjective and highly dependent on experience, and meet the clinical demand for efficient, accurate and automatic methods, Liang et al. [19] proposed the fetal standard section recognition network SPRNet with DenseNet as the backbone network. In order to solve the problems of low identification efficiency and time-consuming manual screening caused by subtle differences between fetal standard sections (FSPs) and non-standard sections in ultrasound videos, Kong et al. [20] proposed a universal detection framework based on multi-scale dense network (MSDNet), which combines multi-scale architecture and dense connection. The recognition efficiency and accuracy are improved. In order to achieve high-precision identification and quality assessment of fetal facial ultrasound standard sections in early pregnancy and early screening of chromosomal abnormalities such as Down syndrome, Xue et al. [21] proposed an integrated scheme to integrate clinical protocols with lightweight target detection. In order to solve the deployment bottleneck problem caused by the large number of SonoNet model parameters in the detection of fetal ultrasound standard sections (FUSPs), Yu et al. [22] proposed a LPC-SonONET network based on lightweight pyramid convolution (LPC). To address the issues of the time-consuming and labor-intensive process of the manual selection of the pubic symphysis–fetal head standard section (PSFHSP), as well as the reliance on the physician’s experience for the results, and to meet the clinical requirements for real-time and precise identification in the measurement of the labor process angle, Qiu et al. [23] proposed the PSFHSP-Net lightweight network based on deep compression optimization.

To solve the clinical problems of high false negative rates and lack of professional doctors in fetal ultrasound imaging diagnosis, and break through the limitation of existing algorithms that can only detect a single part (either abdomen or head), Deepika et al. [24] proposed a two-channel convolutional neural network (tCNN) diagnostic framework for whole fetal abnormalities to achieve a simultaneous diagnosis of normal or abnormal fetal abdominal and fetal brain images. In order to solve the problem of organ misclassification (such as the confusion between kidney and abdomen) caused by the separation of local and global features in fetal ultrasound images, and to excavate new indicators of abnormal development embedded in images in large-scale retrospective studies, Sridar et al. [25] proposed a global–local dual-flow fusion classification framework. By combining global image and local features to classify 2D fetal ultrasound image planes, the automatic classification of 14 different fetal structures was achieved and the classification accuracy was improved. In order to solve the boundary blurring problem caused by strong speckle noise, low soft-tissue contrast and oligohyamnion in ultrasound images in fetal biometry, Oghli et al. [26] proposed the multi-feature pyramid MFP-Unet framework based on attention gating to achieve the accurate segmentation and measurement of four key fetal parameters: biparietal diameter (BPD), head circumference (HC), abdominal circumference (AC) and femur length (FL). In order to solve the risk of misjudgment caused by the prediction uncertainty of a single model in the identification of fetal ultrasound standard sections, and to improve the robustness of the location of key anatomical structures in prenatal screening, Krishna et al. [27] proposed a deep CNN fusion framework based on Stacking Ensemble. Three pre-trained deep CNN models (AlexNet, VGG-19, and DarkNet-19) were combined to make predictions through softmax and random forest classifiers, and the final predictions were determined using the absolute majority voting technique. Avazov et al. [28] proposed a CNN-based method for fetal ultrasound image classification, comparing models like AlexNet and Inception-v3, achieving fast performance without compromising accuracy. Chongwen et al. [29] introduced a semi-supervised segmentation approach using bidirectional prototype-guided consistency constraint (BiPCC), outperforming existing methods in fetal ultrasound segmentation. Linde et al. [30] developed a CNN for 3D fetal brain ultrasound segmentation using few-shot learning, achieving high accuracy with minimal annotations. Netzahualcoyotl et al. [31] proposed a deep learning method for detecting fetal congenital heart defects in three-vessel view ultrasounds, achieving high segmentation and classification performance. Mohammad et al. [32] presented a multi-task learning approach for biometric estimation from fetal ultrasound scans, achieving high accuracy in head circumference and femur length measurements.

## 3. Methodology

This section describes the materials and methodological framework used in this study. First, the construction of the FAUSP dataset and the annotation strategy are introduced. Then, the architecture of the proposed SEG-FAUSP model and its key modules are presented in detail. Finally, the loss function and the experimental settings are described to ensure the reproducibility and reliability of the proposed approach.

### 3.1. Image Annotation

This study collected 4010 ultrasound images of the fetal abdomen from 1765 pregnant women, with a mean age of 33 ± 2 years and a mean body mass index (BMI) of 23.2 ± 2.3 kg/m^2^. All participants were in the mid-pregnancy stage (18–24 weeks). The study was approved by the Institutional Review Boards (IRBs) of the Second Affiliated Hospital of Fujian Medical University and Quanzhou First Hospital (Approval No.: 2024(395)). Written informed consent was obtained from all participants prior to data acquisition. To protect patient privacy, all ultrasound images were anonymized and de-identified before analysis to ensure the security of personal information.The images were acquired using 12 different brands and models of mainstream color Doppler ultrasound diagnostic instruments, including Voluson E8 and Voluson E10 (GE HealthCare, Buckinghamshire, UK), all equipped with 5.0 MHz convex probes capable of real-time fetal heart rate and structural imaging, thereby ensuring data diversity and representativeness.

The inclusion criteria were defined as follows: the target abdominal structure was complete, clear, centered, and occupied at least half of the image area; no extra measurement lines were present except for the standard abdominal circumference cross-section; no obvious noise or artifacts existed; the ultrasound-estimated gestational age was consistent with the actual gestational age; and no structural abnormalities of the fetal abdomen were confirmed by postnatal diagnosis. Images were excluded if they had poor quality, unclear anatomical structures due to fetal position, or detectable malformations.

All images were annotated using Labelme 5.5.0 software for instance segmentation. To ensure reliable annotation, one expert with over ten years of ultrasound experience performed the labeling, while a second expert, also with over ten years of experience, conducted a thorough review. Any discrepancies were resolved through discussion with a third expert, equally experienced, to reach the most accurate and reliable annotation.

Notably, multiple images may be obtained from the same pregnant woman. To eliminate patient-level data leakage, we performed strict patient-independent train–test splitting without overlapping subjects between subsets. The class distribution of the dataset was analyzed, and corresponding strategies were adopted to alleviate data imbalance. The dataset can be made available to other researchers for non-commercial academic use upon reasonable request, following relevant ethical and privacy requirements.

### 3.2. Model Framework

This study proposes the SEG-FAUSP multi-task deep learning model for segmenting key anatomical structures in fetal abdominal ultrasound images, using DeepLabv3+ as the benchmark architecture. The selection of the baseline framework and integrated modules is strictly task-driven by the clinical characteristics and segmentation challenges of fetal abdominal ultrasound imaging. DeepLabv3+ is adopted due to its proven effectiveness in medical image semantic segmentation. Its atrous convolution and encoder–decoder architecture preserve high-resolution feature representations while maintaining large receptive fields, making it particularly suitable for handling the low contrast and noise characteristics of ultrasound images.

To address the major challenges in fetal abdominal ultrasound segmentation—including multi-scale anatomical structures, scattered organ distribution, blurred micro-structure boundaries, and severe ultrasound artifacts—four feature enhancement modules, namely ASPP, MSCA, BiFormer, and CCA, are hierarchically integrated into the baseline architecture.

The theoretical innovation of this module combination lies in a hierarchical feature enhancement pipeline specifically designed for fetal abdominal anatomy. Unlike conventional hybrid networks that simply stack multiple modules, SEG-FAUSP establishes a task-oriented functional matching strategy, where each module is introduced according to the anatomical characteristics and segmentation requirements of fetal abdominal organs. This design enables adaptive feature enhancement tailored to the clinical task. The pipeline sequentially integrates multi-scale feature extraction, long-range dependency modeling, contextual attention interaction, and high–low level feature fusion. Specifically, the encoder first captures coarse multi-scale contextual information using atrous convolution and the ASPP module. Subsequently, MSCA, BiFormer, and CCA are incorporated to progressively refine feature representations from local structural details to global contextual dependencies. Finally, the decoder fuses high-level semantic features with low-level spatial details to generate accurate segmentation masks.

**Figure 1:**
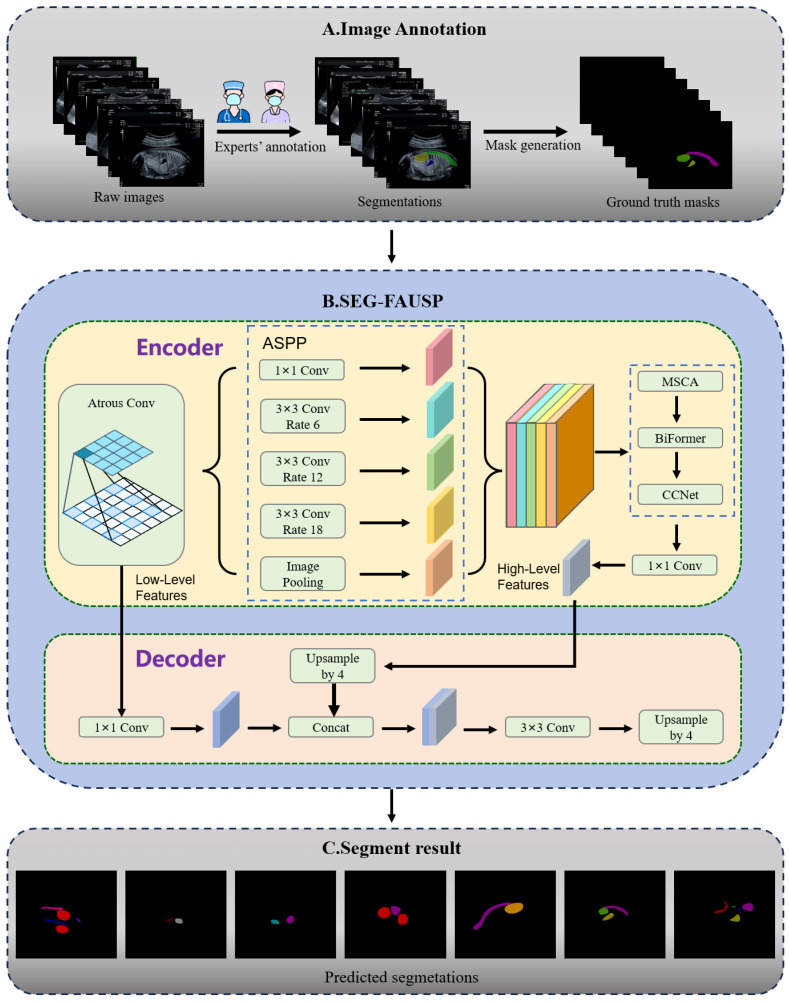
Overall workflow of the proposed SEG-FAUSP framework. First, fetal abdominal ultrasound images are collected and annotated by clinical experts to generate pixel-level masks of key anatomical structures. The images are then used to train the proposed SEG-FAUSP network. The network performs semantic segmentation to identify multiple anatomical structures simultaneously.

As illustrated in Figure 1B, the SEG-FAUSP network follows an encoder–decoder architecture, where the core modules are sequentially embedded within the encoder to form a complementary feature enhancement pipeline. Input ultrasound images first undergo data augmentation and preprocessing, after which they are fed into the backbone network for feature extraction. The neck network performs multi-scale feature aggregation based on the hierarchical enhancement pipeline, and the fused features are then delivered to the segmentation head for precise localization of fetal abdominal organs.

Within the encoder, the modules function collaboratively as follows. Dilated convolution and the ASPP module aggregate contextual information across multiple receptive fields, establishing a robust multi-scale feature representation of fetal abdominal structures such as the spine and umbilical artery inlet. Following ASPP, the MSCA module extracts fine-grained local features across multiple spatial scales and assigns attention weights to salient structures. This mechanism is particularly suitable for capturing elongated anatomical structures such as the umbilical vein as well as organs with varying sizes.

Subsequently, the BiFormer module is introduced to model long-range dependencies among spatially dispersed fetal abdominal organs, including the bilateral kidneys, gastric bubble, and bladder. This component compensates for the limited global contextual modeling capability of MSCA. Finally, the CCA module strengthens contextual attention interaction by computing the affinity matrix between feature positions, enabling enhanced representation of structures with ambiguous boundaries, such as the inferior vena cava.

After the four modules complete hierarchical feature refinement, a 1 × 1 convolution layer is applied for channel compression, producing high-level semantic feature maps. These encoder features are then passed to the decoder to generate the final segmentation results. The decoder first upsamples the high-level features by a factor of four and adjusts channel dimensions using 1 × 1 convolution. The upsampled features are then concatenated with low-level features extracted from the early encoder layers, allowing fine spatial details to be fused with high-level semantic information. A 3 × 3 convolution is applied to refine the fused features, followed by another 4× upsampling step to restore the feature map to the original image resolution. Finally, a 1 × 1 convolution layer produces the predicted segmentation masks for key fetal abdominal anatomical structures (Figure 1C).

The predicted masks are compared with expert-annotated ground truth labels for quantitative evaluation. Extensive experimental validation demonstrates that SEG-FAUSP achieves reliable and accurate segmentation performance for key fetal abdominal anatomical structures, providing a useful technical reference for further research on the automated analysis of fetal abdominal ultrasound images.

#### 3.2.1. ASPP

**Figure 2:**
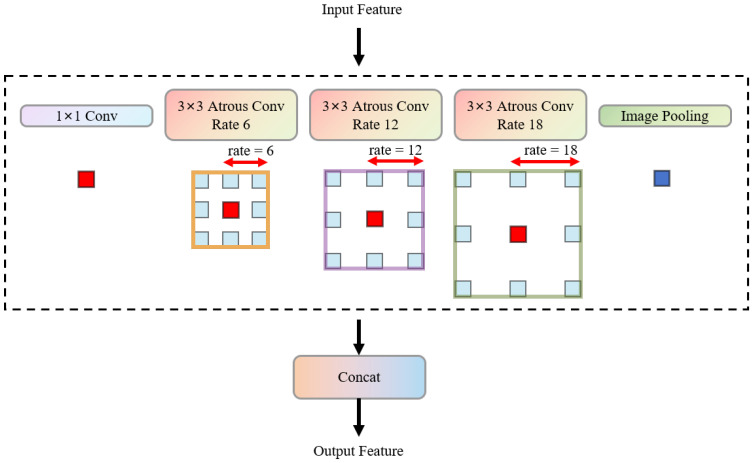
Architecture diagram of ASPP module.

The Figure 2 shows an Atrous Spatial Pyramid Pooling (ASPP) [33] module, which is a key component in deep convolutional neural networks for capturing multi-scale context information. This ASPP module consists of four parallel atrous convolutional layers. Each atrous convolutional layer uses a 3 × 3 convolution kernel, but has different dilation rates (rates). Specifically, these dilation rates are 6, 12, 18, and 24. The dilation rate parameters determine the sampling interval of the convolution kernel when it performs convolution operations on the input feature map. For example, a 3 × 3 convolution kernel with a dilation rate of r is actually equivalent to a regular convolution kernel with a larger receptive field, with an effective receptive field size of (2r + 1) × (2r + 1). Therefore, the four atrous convolutional layers have effective receptive fields of 13 × 13, 25 × 25, 37 × 37, and 49 × 49, respectively, and can capture the context information of different scales in the input feature map without significantly increasing the number of parameters and computational cost. After the input feature map is processed by these four atrous convolutional layers with different dilation rates, the feature maps output by each layer are concatenated together to form a feature representation that integrates multi-scale context information. This multi-scale feature fusion approach helps the model understand the image content more comprehensively, especially for tasks such as semantic segmentation that require global and local context information. In this way, the ASPP module effectively enhances the network’s perception ability for objects of different scales and improves the model’s feature extraction and representation performance in complex scenes.

#### 3.2.2. MSCA

**Figure 3:**
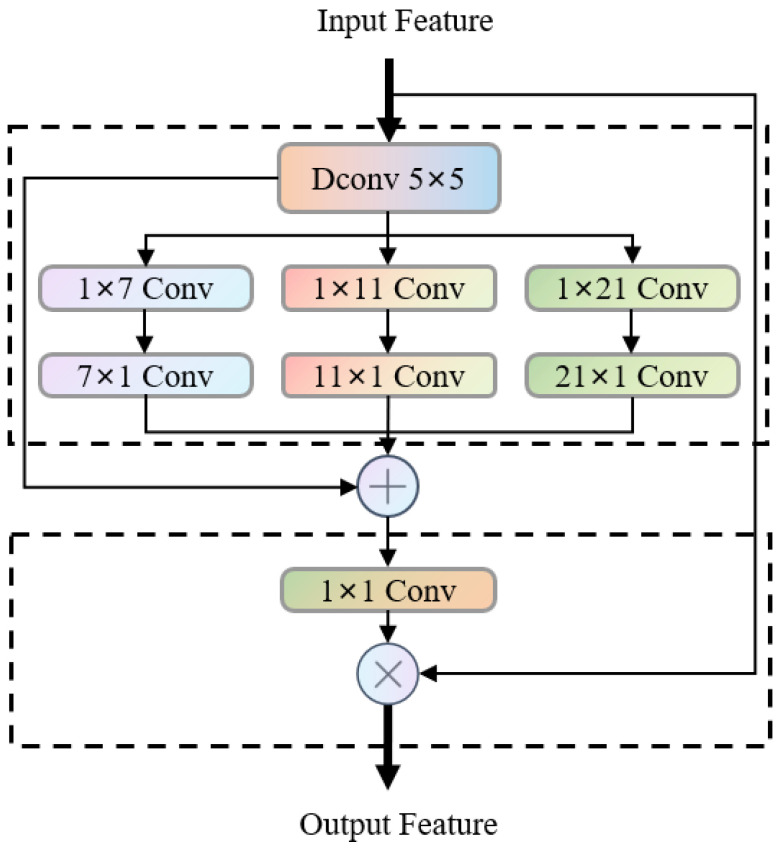
Architecture diagram of MSCA module.

As illustrated in Figure 3, Multi-scale Convolutional Attention (MSCA) [34] is a lightweight multi-scale feature enhancement component. Its core consists of two parts: multi-scale feature extraction and convolutional attention weighting. It is designed to meet the feature capture requirements of different-sized anatomical structures in fetal abdominal ultrasound images.

The MSCA module consists of two core components: Firstly, the input features are aggregated with a 5 × 5 depth convolution (Dconv 5 × 5) to capture local information; subsequently, three parallel branches generate multi-scale features—each branch uses “1 × k + k × 1” orthogonal depth convolution (d is the number of channels), corresponding to equivalent receptive fields of 7 × 7, 11 × 11, 21 × 21, etc., covering the features of small, medium, and large-scale anatomical structures (such as umbilical arteries, bladder, spine). The multi-branch features are fused and dimensionally reduced through channel mixing (1 × 1 convolution) to generate an attention weight map consistent with the input size, and then weighted with the original input features to achieve adaptive focusing on key structures.

MSCA is designed with the “orthogonal depth convolution + multi-branch fusion” approach, achieving multi-scale semantic information coverage with low computational overhead. The strip convolution structure has a targeted extraction advantage for long strip-shaped structures in the fetal abdomen (such as umbilical veins), and can efficiently enhance the spatial resolution of features.

#### 3.2.3. BiFormer

**Figure 4:**
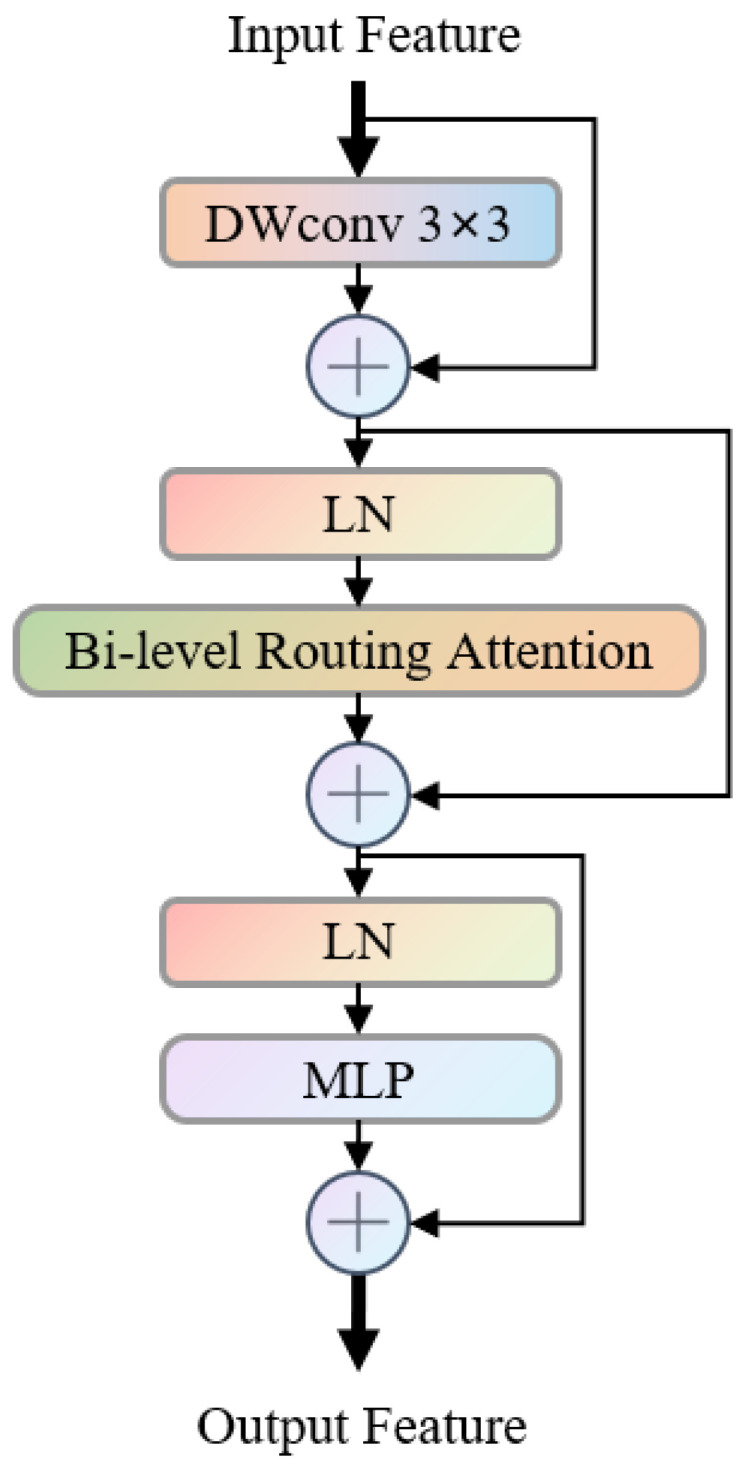
Architecture diagram of BiFormer module.

As illustrated in Figure 4, the Bi-level Routing Attention module (BiFormer) [35] is a long-range dependency modeling component based on residual structures. Its core consists of a sequential structure of “local convolution enhancement + bi-level routing attention + feature transformation”, which achieves efficient global–local feature fusion and meets the modeling requirements for the correlated structures of the fetal abdominal region with its scattered anatomical features.

The specific process is as follows: the input features are first extracted with a 3 × 3 depth convolution (DWConv 3 × 3) to capture local details, then the layer normalization (LN) and residual connection are used to retain the original features; subsequently, it enters the Bi-level Routing Attention module, where regional filtering and token-level calculations capture long-range semantic associations, and then the attention features are fused through layer normalization and residual connection; finally, the multi-layer perceptron (MLP) completes the feature transformation in the channel dimension, and the residual connection is used to output the ultimately enhanced features. BiFormer combines local convolution and bi-level routing attention in a residual structure to control the computational complexity while maintaining local detail retention and long-range structural association modeling. It can effectively improve the feature consistency in the complex anatomical layout of the fetal abdomen.

In the SEG-FAUSP model, BiFormer is embedded in the high-level feature extraction stage of the encoder and forms complementarity with MSCA: MSCA focuses on multi-scale local features and spatial attention guidance, while BiFormer is responsible for long-range anatomical structure association modeling, jointly enhancing the feature representation ability of the complex anatomical layout of the fetal abdomen.

#### 3.2.4. CCA

**Figure 5:**
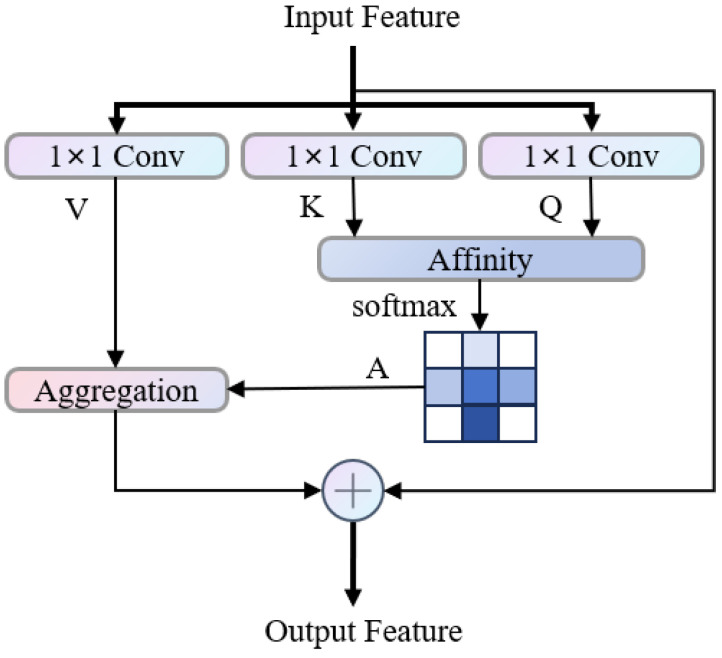
CCA module architecture diagram.

The Figure 5 shows the workflow of the CCA [36] module. The three-dimensional block labeled “H” on the left is the input feature of the module. It is first processed by three parallel 1 × 1 convolutions to obtain Query (Q), Key (K), and Value (V). This operation maps the original features to different spaces to extract corresponding feature representations. Then, using Q and K, the dot product similarity is calculated and normalized by softmax to obtain the affinity matrix A. The elements in A measure the degree of attention at different positions and reflect the semantic or feature correlation between positions. Finally, the affinity matrix A is used to weight and aggregate the Value branch to obtain the enhanced feature H’, where each position references the affinity weight and combines the relevant features in V, making the output feature take into account both local details and long-distance context dependencies. This module achieves efficient long-distance feature modeling and controls the computational complexity through the above process, improving the feature expression ability in visual tasks.

### 3.3. Loss Function

In the semantic segmentation task of fetal abdominal ultrasound images in this study, there are challenges such as significant differences in the sizes of multiple anatomical structures and extremely unbalanced pixel distribution. For instance, the pixels of micro-anatomical structures like the umbilical artery entrance and the inferior vena cava account for a very low proportion, while the pixels of large structures such as the spine and abdominal circumference account for a significantly higher proportion. This greatly increases the difficulty of the automated segmentation task. Although the traditional cross-entropy loss [37] can effectively measure the classification accuracy of the model for various categories, it is prone to cause the model to favor predicting the majority category with a higher pixel proportion and ignore the minority category with a lower pixel proportion, resulting in a significant decline in the segmentation accuracy of micro-anatomical structures and failing to meet the clinical requirements for fine segmentation of fetal abdominal structures.

To address this issue, this study introduces CEDice Loss as the loss function of the model. This loss function combines the advantages of cross-entropy loss in class classification and Dice Loss [38] in measuring the overlap degree of regions, and can simultaneously take into account the classification accuracy of pixel categories and the regional segmentation integrity of anatomical structures. It effectively alleviates the segmentation deviation caused by class imbalance, especially improving the segmentation performance of micro-anatomical structures.

The core design idea of CEDice Loss is to weight and fuse the cross-entropy loss and the Dice Loss. It retains the accuracy of pixel-level classification of the cross-entropy loss and constrains the overlap degree of the predicted region and the true labeled region through the Dice Loss, allowing the model to simultaneously focus on the pixel classification results and the segmentation effect of the structural region during training. Its calculation formula is as follows:

(1) CEDiceLoss=α·CE+(1−α)·(1−Dice)

Among them, CE represents cross-entropy loss, and its calculation formula is:

(2) $$\mathrm{CE}=-\sum_{i=1}^{n}\log Q\left(i\right)\cdot P\left(i\right)$$

Dice is the Dice coefficient, which is used to measure the degree of overlap between the predicted segmented area and the actual segmented area. The calculation formula is:

(3) $$\mathrm{Dice}=\frac{2\cdot |A\cap B|}{\left|A|+|B\right|}$$

Among them, Q(i) and P(i) respectively represent the probability distributions of the model’s predicted output and the gold standard annotation; *A* is the segmented area predicted by the model, *B* is the true segmented area annotated by the expert, |A∩B| is the number of pixels in the intersection of the two areas, and |A| and |B| are the total pixel numbers of the predicted area and the true area respectively; α is the weighting coefficient. In this study, α is set to 0.5 to achieve the balanced constraint of cross-entropy loss and Dice Loss, and it can be adaptively adjusted according to the requirements of different segmentation tasks.

## 4. Experiments and Results

This section presents the experimental setup, results, and detailed analysis of the proposed SEG-FAUSP model’s performance on fetal abdominal ultrasound image segmentation. The experiments were designed to evaluate the effectiveness of our model in various aspects, including segmentation accuracy and computational efficiency. The subsequent subsections provide detailed insights into the experimental configuration and the performance of the model.

### 4.1. Experimental Setup

**Figure 6:**
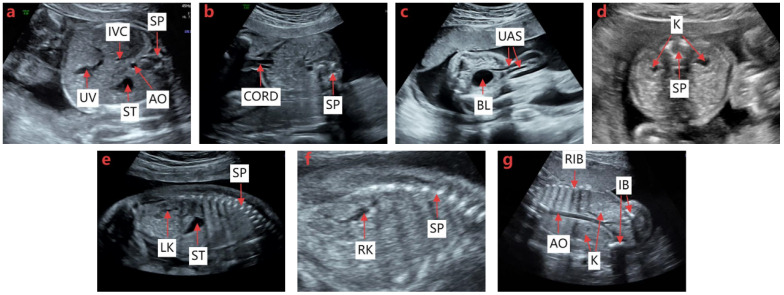
Seven FAUSP images. (**a**) ACS; (**b**) UCS; (**c**) BHS; (**d**) BKHS; (**e**) LKS; (**f**) RKS; (**g**) BKCS.

As illustrated in Figure 6, we used a dataset of 4010 ultrasound images covering seven fetal abdominal standard planes, which contains 629 images for ACS, 804 images for UCS, 366 images for BHS, 497 images for BKHS, 682 images for LKS, 614 images for RKS, and 418 images for BKCS. To perform the semantic segmentation task, the FAUSP dataset was randomly divided into training, validation, and test sets with each standard plane accounting for 8:1:1 (80%, 10%, and 10%, respectively). To further evaluate the robustness and generalization ability of the proposed model under limited dataset size, a five-fold cross-validation strategy was additionally adopted during training. In this strategy, the dataset was divided into five subsets, and each subset was used once as the validation set while the remaining subsets were used for training. The final performance was obtained by averaging the results of all folds. The experiments were performed using a 12-generation Intel(R) Core(TM) i5-12400F CPU and an NVIDIA GeForce RTX3050 GPU equipped with 16 GB of video memory. All experiments were conducted on a computer running a 64-bit Windows 10 operating system. The model was implemented in Python 3.8 using PyCharm 2025.2.3 ×64. The network was trained for 120 epochs with the SGD optimizer. The initial learning rate was set to 0.01 dated using a cosine annealing learning rate schedule. To enhance model generalization and reduce overfitting, data augmentation techniques including random flipping, rotation, and scaling were applied during training.

### 4.2. Evaluation Metrics

To evaluate the segmentation accuracy of the model, we selected eight commonly used evaluation metrics in semantic segmentation tasks, including pixel accuracy (PA) [39], Dice coefficient [40], and intersection over union (IoU) [41], average surface distance [42], nean pixel accuracy (mPA), mean Dice coefficient (mDice), mean intersection over union (mIoU), and Mean Average Surface Distance (mASD). Pixel accuracy measures the proportion of pixel values predicted by the model that are correctly predicted. The Dice coefficient measures the degree of overlap between the segmentation region predicted by the model and the true segmentation region, and is calculated as the intersection area of twice divided by the total number of pixels of the two regions. The intersection over union ratio is also a classical index to measure the overlap degree of the model. Its value is the intersection area of the predicted region and the true region divided by their union area. Average surface distance is used to measure the boundary accuracy of segmentation results. The average pixel accuracy (PA) is calculated as the arithmetic mean of the pixel accuracy (PA) of all classes, which can reflect the overall classification accuracy of the model more comprehensively than the PA of a single class. The average Dice coefficient is calculated as the average of all categories, which can comprehensively evaluate the overall segmentation performance. The mean intersection over union (mIoU) is the average IoU of all classes. Like mDice, it is a commonly used index to evaluate the region overlap degree of multi-class segmentation models. Mean Average Surface Distance is calculated as the average of all categories of ASD, which is used to comprehensively evaluate the average accuracy of the model in segmenting boundaries on all categories.

TP (true positive) means the actual class is positive and the predicted class is positive. FP (false positive) means the actual class is negative and the predicted class is positive. TN (true negative) means the actual class is negative and the predicted class is negative. An FN (false negative) means the actual class is positive class and the predicted class is negative. S(A) represents the surface voxel in the predicted segmentation region A, and d (V, S(A)) represents the shortest distance from any voxel to the predicted segmentation region A. S(B) represents the surface voxel in the true segmentation region B, and d (V, S(B)) represents the shortest distance from any voxel to the predicted segmentation region B. The higher the values of PA, Dice and IoU indexes, the more accurate the segmentation results are. A smaller value of ASD indicates better segmentation performance.

(4) $$\mathrm{PA}=\frac{\mathrm{TP}+\mathrm{TN}}{\mathrm{TP}+\mathrm{TN}+\mathrm{FP}+\mathrm{FN}}$$

(5) $$\mathrm{Dice}=\frac{2\mathrm{TP}}{2\mathrm{TP}+\mathrm{FP}+\mathrm{FN}}$$

(6) $$\mathrm{IoU}=\frac{\mathrm{TP}}{\mathrm{TP}+\mathrm{FP}+\mathrm{FN}}$$

(7) $$\mathrm{ASD}=\frac{1}{S(A)+S(B)}\left(\sum_{s_{A}\in S\left(A\right)}d\left(s_{A},S\left(B\right)\right)+\sum_{s_{B}\in S\left(B\right)}d\left(s_{B},S\left(A\right)\right)\right)$$

(8) $$\mathrm{mPA}=\frac{\mathrm{TP}+\mathrm{TN}}{n(\mathrm{TP}+\mathrm{TN}+\mathrm{FP}+\mathrm{FN})}$$

(9) $$\mathrm{mDice}=\frac{2\mathrm{TP}}{n(2\mathrm{TP}+\mathrm{FP}+\mathrm{FN})}$$

(10) $$\mathrm{mIoU}=\frac{\mathrm{TP}}{n(\mathrm{TP}+\mathrm{FP}+\mathrm{FN})}$$

(11) $$\mathrm{mASD}=\frac{1}{n(S(A)+S(B))}\left(\sum_{s_{A}\in S\left(A\right)}d\left(s_{A},S\left(B\right)\right)+\sum_{s_{B}\in S\left(B\right)}d\left(s_{B},S\left(A\right)\right)\right)$$

### 4.3. Experiment Results

This section provides a comprehensive overview of the results obtained from the experiments. We analyze the performance of the SEG-FAUSP model in terms of segmentation accuracy, including comparison with baseline models. Furthermore, we present detailed performance metrics and evaluate the model’s ability to generalize across various anatomical structures.

#### 4.3.1. Semantic Segmentation Results

**Figure 7:**
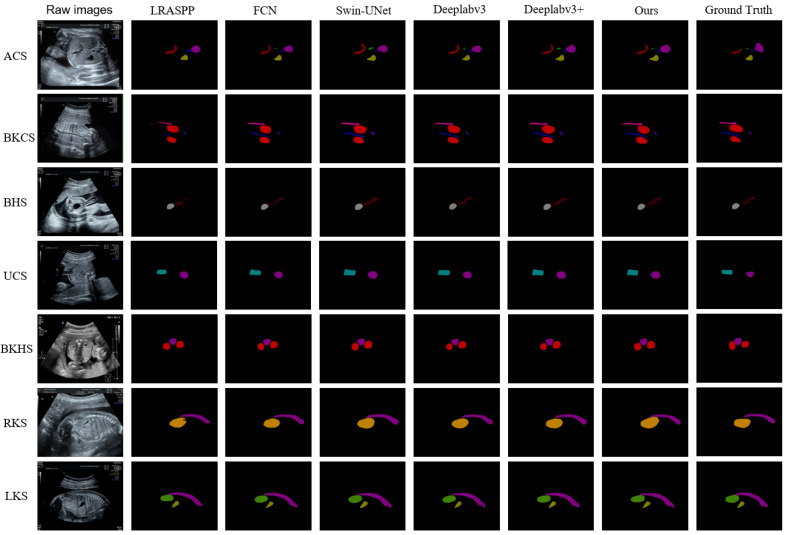
Visualization of segmentation results under different models.

Based on the SEG-FAUSP model proposed in this paper, we achieved semantic segmentation of the organs in FAUSP images. Figure 7 visually illustrates the segmentation differences of fetal abdominal ultrasound anatomical structures between SEG-FAUSP and the comparison models, and clearly demonstrates the superiority of the proposed model in boundary integrity and structural localization accuracy. Table 1 presents a comparative overview of the segmentation performance of each anatomical structure organ. We compared the model with LRASPP, DeepLabv3 [33], DeepLabv3+ [43], FCN, and Swin-UNet [44]. The experimental results are shown in Table 1, indicating that Deeplabv3+ is the best model for this task, proving that this model is generally more accurate than traditional convolutional neural network models. Our SEG-FAUSP model is an improved version based on Deeplabv3+. Table 2 shows the quantitative evaluation results of the segmentation performance of the SEG-FAUSP model for the key anatomical structures of the fetal abdomen. The table lists 13 anatomical structures including umbilical vein (UV), inferior vena cava (IVC), gastric bubble (ST), abdominal aorta (AO), spine (SP), umbilical cord (CORD), bladder (BL), umbilical artery inlet (UAS), kidney (K), left kidney (LK), right kidney (RK), ilium (IB), and ribs (RIB). The intersection over union (IoU), Dice coefficient, pixel accuracy (PA), and average surface distance (ASD) indicators are listed. From the data, it can be seen that the segmentation accuracy of structures such as ST (IoU = 0.842) and BL (IoU = 0.844) is relatively high, while structures such as IVC (IoU = 0.546) and UAS (IoU = 0.572) perform relatively poorly due to their complex morphology or low contrast. This table verifies the robustness of the model in multi-structure segmentation tasks and provides a basis for subsequent section recognition.

**Figure 8:**
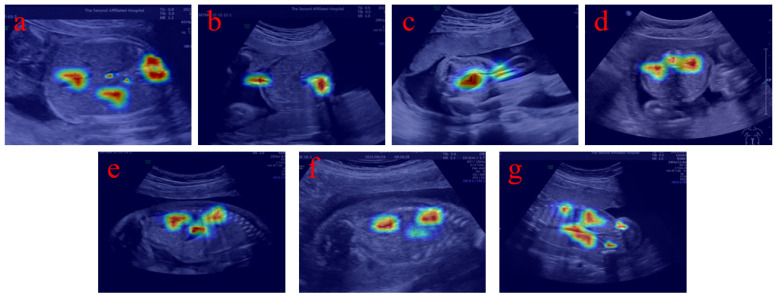
Representative FAUSP images and visualization of model interpretability. (**a**) ACS; (**b**) UCS; (**c**) BHS; (**d**) BKHS; (**e**) LKS; (**f**) RKS; (**g**) BKCS. The corresponding heatmaps illustrate the regions that contribute most to the model predictions, showing that the network primarily focuses on key anatomical structures.

To further enhance the interpretability of the proposed model, we additionally provide visualization results based on heatmaps to illustrate how the network focuses on relevant anatomical regions during prediction. As shown in Figure 8, the highlighted regions in the heatmaps are highly consistent with the locations of key anatomical structures such as the gastric bubble, spine, and umbilical vein. This indicates that the model is able to effectively capture clinically meaningful features rather than relying on irrelevant background information. These visual explanations provide intuitive evidence that the model decision process aligns with clinical knowledge, thereby improving the transparency and reliability of the proposed method in medical applications.

In addition to accuracy, we also evaluated the inference speed by measuring the frames per second (FPS) of each model to assess its feasibility for real-time clinical applications. The SEG-FAUSP model achieved an FPS of 3.723, which is competitive with models like DeepLabv3+ (FPS = 4.012). This result underscores the SEG-FAUSP model’s ability to balance segmentation accuracy with processing speed, making it suitable for real-time ultrasound analysis, which is critical in clinical settings for efficient and timely diagnoses.

#### 4.3.2. Ablation Experiments

To systematically evaluate the contribution of each component in the proposed SEG-FAUSP architecture, an ablation study was conducted on the FAUSP dataset. Table 3 presents the ablation results for the four components (CEDice Loss, MSCA, BiFormer, and CCA). Starting from the baseline DeepLabV3+ model, these modules were gradually introduced to analyze their individual contributions to segmentation performance. This experiment uses DeepLab v3+ as the baseline model. By gradually introducing each component, the contribution of these components to the segmentation performance of fetal abdominal ultrasound images is evaluated. The evaluation metrics include mean intersection over union (mIoU), mean Dice coefficient (mDice), mean pixel accuracy (mPA), and mean surface distance (mASD). The mIoU of the baseline model (without introducing any new components) is 0.717, mDice is 0.829, mPA is 0.820, and mASD is 0.282. When only CEDice Loss is introduced, all the precision indicators of the model improve: mIoU rises to 0.733, mDice rises to 0.841, mPA rises to 0.855, and mASD drops to 0.267. This indicates that CEDice Loss can effectively optimize the loss calculation in the segmentation task and significantly improve the segmentation accuracy and the refinement of boundary segmentation effects. After adding the MSCA module on top of introducing CEDice Loss, the indicators further improve: mIoU reaches 0.734, mDice reaches 0.842, mPA reaches 0.858, and mASD drops to 0.265. This shows that the MSCA module can effectively capture multi-scale feature information and enhance the model’s ability to segment different-scale anatomical structures. Continuing to add the BiFormer module, the model performance continues to improve: mIoU increases to 0.735, mDice increases to 0.843, mPA increases to 0.859, and mASD further drops to 0.264. This demonstrates that the bidirectional feature fusion mechanism of BiFormer can more fully integrate context information, further improving the accuracy and completeness of segmentation.

When all four components are introduced, the model achieves the best performance in all indicators: mIoU reaches 0.741, mDice reaches 0.847, mPA reaches 0.866, and mASD drops to the lowest 0.261. This indicates that the collaborative effect of CEDice Loss, MSCA, BiFormer, and CCA can simultaneously improve performance from four dimensions: loss optimization, multi-scale feature extraction, bidirectional feature fusion, and context attention modeling. This effectively addresses the segmentation difficulties in fetal abdominal ultrasound images and significantly improves segmentation accuracy and boundary integrity.

## 5. Discussion

This study addresses the clinical challenges such as the reliance on physician experience for fetal abdominal ultrasound standard plane (FAUSP) identification and the interference of ultrasound artifacts on diagnostic accuracy. It proposes a deep learning-based multi-task segmentation model, SEG-FAUSP, which achieves accurate segmentation of seven types of key anatomical structures. This provides a potential computational tool for assisting the analysis of fetal abdominal ultrasound images.

The experimental results show that the SEG-FAUSP model exhibits excellent robustness in 13 core anatomical structure segmentation tasks. The segmentation accuracy of gastric bubble (ST, IoU = 0.842) and bladder (BL, IoU = 0.844) is particularly outstanding, thanks to the regular shape and high contrast of these structures in ultrasound images. This also verifies the model’s ability to accurately capture clear anatomical targets. However, for complex structures such as inferior vena cava (IVC, IoU = 0.546) and umbilical artery entrance (UAS, IoU = 0.572), the segmentation performance is relatively limited. The main reason is that the diameter of IVC is thin and the boundary with surrounding tissues is blurry. The anatomy of UAS is concealed and the morphology varies greatly. Moreover, the image quality fluctuation caused by ultrasound artifacts increases the difficulty of feature extraction. However, compared to the baseline model Deeplabv3+, the segmentation accuracy of SEG-FAUSP for these complex structures has still improved by 3.2–5.7%. This improvement is attributed to the collaborative effect of the model’s multi-module fusion: the MSCA module efficiently captures the feature information of long strip-like structures (such as spine and umbilical vein) through multi-scale strip convolution, the BiFormer module precisely models the long-distance dependencies of dispersed organs (such as both kidneys, gastric bubble and bladder) with a dual-level routing attention mechanism, and the CCA module strengthens the interaction and fusion of contextual features. The combination of these three modules with the CEDice Loss enhances the segmentation performance from the three dimensions of feature extraction, dependency modeling and loss optimization. This conclusion is also fully verified by ablation experiments—when all four components are introduced, the model’s mIoU reaches 0.741, an increase of 3.3% compared to the baseline model, and mASD drops to 0.261, significantly improving the boundary segmentation accuracy.

Beyond the superior segmentation performance validated by experiments and ablation studies, this work presents distinctive innovations compared with existing hybrid segmentation networks and relevant research in the field, mainly in three aspects. First, the SEG-FAUSP architecture adopts a task-specific module selection and hierarchical complementary coupling strategy: unlike conventional networks that integrate mainstream modules randomly or in simple parallel (which easily causes feature redundancy and high computational complexity), we select and combine ASPP, MSCA, BiFormer and CCNet strictly based on the clinical characteristics and segmentation pain points of fetal abdominal ultrasound, and arrange them into a sequential hierarchical pipeline. The modules complement each other in local multi-scale extraction, global long-range modeling and contextual attention interaction, forming a synergistic feature enhancement effect tailored for fetal abdominal anatomy. Second, the model realizes lightweight and adaptive integration of Transformer and CNN modules: different from existing Transformer–CNN hybrid networks that introduce heavy Transformer blocks leading to poor clinical deployability, SEG-FAUSP embeds the lightweight BiFormer module into the encoder and combines it with CNN-based modules via a residual structure. This design controls computational costs while boosting global feature modeling capability, making the model suitable for deployment in primary healthcare institutions with limited computing resources. Third, we break through the limitations of existing research in data and framework design: a standardized dataset with 4010 images covering seven clinical standard planes and various mainstream ultrasound devices is constructed, filling the gap of high-quality labeled data for fetal abdominal ultrasound multi-plane segmentation; meanwhile, an integrated end-to-end “segmentation-derivation” framework is proposed, which directly infers slice types from anatomical structure segmentation results and abandons the traditional stepwise “feature extraction-classification recognition” process, reducing reliance on large-scale pre-trained data and achieving higher clinical practicality and data efficiency than existing relevant models.

The clinical value of this study is mainly reflected in the following. Through automated segmentation of key anatomical structures, it provides an objective basis for the interpretation of standard slices, effectively reducing diagnostic differences caused by the operation of physicians with different levels of experience. It is particularly suitable for scenarios where there is a shortage of ultrasound professionals in grassroots medical institutions. The model’s precise segmentation of the core structures in the cross-sectional plane of the abdominal circumference provides data support for fetal growth and development assessment (such as abdominal circumference measurement), and can also assist in indicating abnormal conditions such as permanent right umbilical vein and esophageal atresia. These segmentation results may provide objective structural references for clinicians and could assist in the interpretation of ultrasound standard planes. However, the current study only evaluates segmentation performance, and further clinical validation and reader studies are required to determine its potential impact on diagnostic accuracy.

However, this study still has certain limitations: Firstly, although the dataset covers various ultrasound devices, the samples mainly come from a single regional medical institution, and the geographical representativeness is limited. In the future, it is necessary to expand the geographical distribution and population diversity of the samples to further improve the generalization ability of the model. Secondly, the current model is only optimized for mid-pregnancy (18–24 weeks) fetal abdominal ultrasound images. The adaptability of the model for early pregnancy (11–13+6 weeks) fetal organs that have not fully developed and late pregnancy (28–36 weeks) fetal body position creates limitations requiring image feature changes to be verified. In addition, in extreme cases with extremely poor ultrasound image quality (such as severe artifacts and insufficient amniotic fluid), there is still room for improvement in the model’s segmentation accuracy of tiny anatomical structures.

Future research can be conducted in three directions: firstly, expand the coverage of gestational weeks in the dataset to include early pregnancy (11–13 + 6 weeks) and late pregnancy (28–36 weeks) ultrasound images to optimize the model’s adaptability to different gestational weeks’ fetal anatomical features; secondly, introduce cross-modal data fusion strategies, combined with other medical imaging data such as MRI, to improve the segmentation and diagnostic accuracy of complex malformation cases; thirdly, develop a mobile version of the model to simplify the operation process and further lower the usage threshold for grassroots medical institutions, promoting the popularization and application of prenatal screening technology.

In summary, the SEG-FAUSP model achieves precise segmentation of key fetal anatomical structures in abdominal ultrasound through multi-module collaborative optimization, providing an objective and efficient solution for standard section identification. It has the potential to assist ultrasound image analysis and may contribute to improving the standardization of fetal abdominal ultrasound examination. Nevertheless, further multi-center clinical validation studies are required to assess its practical value in clinical applications.

## 6. Conclusions

This study proposes SEG-FAUSP, a multi-task deep learning model for automated segmentation of key anatomical structures in fetal abdominal ultrasound standard planes, assisting automated analysis of fetal abdominal ultrasound images. Experimental results demonstrate that SEG-FAUSP accurately segments 13 anatomical structures across seven standard planes, improving both segmentation precision and diagnostic efficiency. It provides a practical tool for clinical diagnosis, particularly in primary healthcare settings. However, the model’s performance is constrained by dataset scale and fetal gestational age coverage. Future work will further evaluate the proposed model using external datasets and multi-center clinical data to validate its generalization capability. In addition, future studies will explore the automatic recognition of standard fetal ultrasound planes. In summary, SEG-FAUSP offers a reliable foundation for intelligent fetal abdominal ultrasound analysis, with substantial clinical value and potential for further refinement.

## Figures and Tables

**Table 1:** Comparison of the segmentation results of FAUSP obtained by the traditional convolutional neural network method and the SEG-FAUSP proposed by us. The most effective ones are indicated by bold font.

Algorithm	mIoU	mDice	mPA	mASD	FPS
LRASPP	0.573	0.704	0.686	0.427	3.225
FCN	0.705	0.819	0.789	0.295	3.386
Swin-UNet	0.712	0.826	0.802	0.292	3.661
Deeplabv3	0.712	0.824	0.809	0.288	3.568
Deeplabv3+	0.717	0.829	0.820	0.282	**4.012**
Proposed	**0.741**	**0.847**	**0.866**	**0.261**	3.723

**Table 2:** Segmentation results of FAUSP using SEG-FAUSP: IoU, PA, Dice, and ASD.

Class	IoU	Dice	PA	ASD
UV	0.717	0.835	0.870	0.283
IVC	0.546	0.706	0.721	0.454
ST	0.842	0.914	0.912	0.158
AO	0.775	0.873	0.900	0.225
SP	0.755	0.861	0.862	0.245
CORD	0.736	0.848	0.859	0.264
BL	0.844	0.915	0.949	0.156
UAS	0.572	0.727	0.726	0.428
K	0.717	0.835	0.872	0.283
LK	0.735	0.848	0.828	0.265
RK	0.716	0.834	0.875	0.284
IB	0.649	0.787	0.774	0.351
RIB	0.724	0.840	0.899	0.276

**Table 3:** Ablation study of different components in the proposed SEG-FAUSP network. The most effective ones are indicated by bold font.

CEDice Loss	MSCA	BiFormer	CCA	mIoU	mDice	mPA	mASD
Baseline (Deeplab v3+)				0.717	0.829	0.820	0.282
✓				0.733	0.841	0.855	0.267
✓	✓			0.734	0.842	0.858	0.265
✓	✓			0.734	0.842	0.858	0.265
✓	✓	✓		0.735	0.843	0.859	0.264
✓	✓		✓	0.737	0.845	0.861	0.263
✓	✓	✓	✓	**0.741**	**0.847**	**0.866**	**0.261**

## Data Availability

The data are not accessible to the public owing to patient privacy concerns. If you have any research needs, you can contact the corresponding author for assistance.
